# The Effect of Aging on Muscular Dynamics Underlying Movement Patterns Changes

**DOI:** 10.3389/fnagi.2016.00309

**Published:** 2016-12-21

**Authors:** Carlijn A. Vernooij, Guillaume Rao, Eric Berton, Frédérique Retornaz, Jean-Jacques Temprado

**Affiliations:** ^1^Aix Marseille University, CNRS, ISMMarseille, France; ^2^Centre Gérontologique DépartementalMarseille, France

**Keywords:** functional connectivity dynamics, muscle synergy, Fitts' task, dynamical regime, coordination patterns

## Abstract

**Introduction:** Aging leads to alterations not only within the complex subsystems of the neuro-musculo-skeletal system, but also in the coupling between them. Here, we studied how aging affects functional reorganizations that occur both within and between the behavioral and muscular levels, which must be coordinated to produce goal-directed movements. Using unimanual reciprocal Fitts' task, we examined the behavioral and muscular dynamics of older adults (74.4 ± 3.7 years) and compared them to those found for younger adults (23.2 ± 2.0 years).

**Methods:** To achieve this objective, we manipulated the target size to trigger a phase transition in the behavioral regime and searched for concomitant signatures of a phase transition in the muscular coordination. Here, muscular coordination was derived by using the method of muscular synergy extraction. With this technique, we obtained functional muscular patterns through non-negative matrix factorization of the muscular signals followed by clustering the resulting synergies.

**Results:** Older adults showed a phase transition in behavioral regime, although, in contrast to young participants, their kinematic profiles did not show a discontinuity. In parallel, muscular coordination displayed two typical signatures of a phase transition, that is, increased variability of coordination patterns and a reorganization of muscular synergies. Both signatures confirmed the existence of muscular reorganization in older adults, which is coupled with change in dynamical regime at behavioral level. However, relative to young adults, transition occurred at lower index of difficulty (ID) in older participants and the reorganization of muscular patterns lasted longer (over multiple IDs).

**Discussion:** This implies that consistent changes occur in coordination processes across behavior and muscle. Furthermore, the repertoire of muscular patterns was reduced and somewhat modified for older adults, relative to young participants. This suggests that aging is not only related to changes in individual muscles (e.g., caused by dynapenia) but also in their coordination.

## Introduction

The neuro-musculo-behavioral system is composed of multiple interacting subsystems (i.e., cognitive, neural, somatosensory, and muscular). Accordingly, aging is a dynamic process that presents itself with both alterations within all subsystems—though each having their individual timescale—and with changes in their couplings (e.g., Sleimen-Malkoun et al., 2014). These changes lie at the origin of an age-related loss of behavioral adaptability. Thus, understanding how aging affects functional reorganizations that occur both within and between the different subsystems that must be coordinated to produce complex movements is an important challenge for research in gerontology.

Concerning the functional reorganizations that occur within the different subsystems, some of these issues have been addressed in the aging literature. For instance, it has been shown that, besides structural alterations in the brain with aging (e.g., lesser corticostriatal white matter connections or a decrease in gray matter volume) (Babcock and Salthouse, 1990; Kennedy and Raz, 2009; Forstmann et al., 2011), brain activation patterns reorganize toward a dedifferentiation of cognitive and motor areas (Heuninckx et al., 2005, 2008) and links between brain areas become less flexible (Reuter-Lorenz and Cappell, 2008; Park and Reuter-Lorenz, 2009). This suggests an increased locking of informational connections. Regarding the age-related reorganization of the muscular subsystem, a growing body of research reported modifications of both structure and functional activation of muscles, captured by dynapenia. Dynapenia refers to the loss of muscle strength with aging due to mechanisms including both peripheral changes leading to a decrease in muscle mass and changes that occur at central and spinal levels. Together, this results in a diminished maximum voluntary contraction magnitude (Clark and Manini, 2012; Manini and Clark, 2012; Mitchell et al., 2012; Russ et al., 2012) and a decrease in rapid force production capacity (Clark and Manini, 2012; Manini and Clark, 2012; Mitchell et al., 2012; Russ et al., 2012). In addition, studies also often reported co-activation of muscles and redistribution of functional activations over the different muscles involved in the specific action systems (e.g., gait). These phenomena are considered at the origin of the loss of behavioral adaptability observed in older adults. However, little is known about how the aging neuro-muscular system accommodates alterations in its muscular components. A main reason is that, until recently, methods were lacking: (i) to identify muscular coordination patterns among multiple muscles, and (ii) how they evolve over time during movement execution and increase in task constraints. To our knowledge, only one study explored this issue (Monaco et al., 2010). In this study, the authors extracted muscle synergies from young and elderly adults during locomotion at different cadences with muscle synergies being described as a modular organization of muscular activation patterns, as it is classically followed in the studies by the group of d'Avella and Bizzi (seminal paper: d'Avella et al., 2003). Monaco found a similar repertoire of synergy patterns for both age groups. However, whether aging affects reorganizations and/or the nature and flexibility of muscular coordination (during a controlled task) in relationship to changes in the behavioral regime has not been determined. The present study is a step in this direction. To achieve this objective, we capitalized both on the framework of dynamical systems theory and on two methods (muscular synergies and functional connectivity dynamics) during tasks of variable difficulty.

To address this issue, a possible strategy consists of forcing the system to reorganize to accommodate a progressive increase in task constraints (Haken et al., 1985; Kelso and Jeka, 1992; Kelso, 2009; Sleimen-Malkoun et al., 2014). In the present experiment, we adopted a similar strategy to study reorganizations in muscular dynamics in parallel to (before, around, and after) transitions between dynamics regimes in Fitts' task. This strategy has been used in a previous study on the relationship between muscles and behavior in young subjects (Vernooij et al., 2016). Using Fitts' task, by increasing task difficulty via the manipulation of target width, behavior was guided through a phase transition between two dynamical regimes. In parallel, the corresponding muscular coordination of 12 muscles was tracked to assess both functional coordination patterns and the variability of their functional connectivity (De Marchis et al., 2013; Hansen et al., 2015). Note that in our previous study, we do not calculate muscle synergies (“building blocks”) along the line of d'Avella and Bizzi, but solely use the methodology of muscle synergy extraction as part of our analysis to calculate flexibly assembled coordination patterns. Our results have shown parallel reorganizations at muscular and behavioral levels when switching between dynamical regimes. More importantly, we detected typical signatures of a transition at the muscular level; (i) a reorganization of muscular coordination; and (ii) a peak in the variability of muscular coordination patterns. These signatures lend credence to our hypothesis that consistent changes occur between muscle and behavioral subsystems. However, the effects of aging on muscular and behavioral dynamics are unknown. The present study addresses this issue.

A first objective of the present experiment is to determine: (i) whether the two dynamical regimes observed in cyclical Fitts' task persist in older adults and (ii) whether a transition between them occurs (whether at lower or similar difficulty level). Despite the scarce amount of studies available on aging using this approach, several predictions can be made regarding the effect of aging on phase transitions at behavioral and muscular levels. For the behavioral level, two contradictory hypotheses can be put forward based on previous studies in Fitts' and bimanual coordination tasks. First, we could expect to observe a reduced repertoire of dynamical regimes, as observed in a discrete Fitts' task by Sleimen-Malkoun et al. (2013). Specifically, by increasing task difficulty via the manipulation of target width, they found a decrease in the number of patterns in the behavioral repertoire used by elderly. An alternative hypothesis is to observe a comparable dynamics—i.e., similar patterns and a phase transition between them—in young and older adults, though differently modulated by task difficulty. As an illustration, Temprado et al. have studied the effect of aging on bimanual coordination dynamics, that is, on the phase transition between in-phase and anti-phase coordination patterns (Temprado et al., 2010). They found similar dynamics in young and older adults. However, in older adults a phase transition occurred at a lower level of difficulty (i.e., lower movement frequency).

A second objective of this study is to examine the effect of aging on muscular coordination and how muscular dynamics are coupled to behavioral dynamics. Our general prediction is that both dynamics should be closely related in older adults, as in young participants. Accordingly, regarding the first typical signature of a phase transition at the muscular system—reorganization of muscular coordination—in line with the behavioral hypothesis we expected to find either a reduced repertoire of muscular coordination (a smaller number of muscular coordination patterns) and/or a different assembly of the same patterns around any behavioral transition. To test this hypothesis, we will apply a non-negative matrix factorization (NNMF; Tresch et al., 2006) to small time-bins of EMG activity of multiple muscles (comparable to De Marchis et al., 2013) and track the repertoire of the resulting synergies. In this context, according to the framework of dynamical systems theory, muscular synergies extracted through the NNMF are considered as temporarily assembled functional units (Turvey, 2007). Regarding the second signature of a phase transition (i.e., a peak in the coordinative variability), we will use functional connectivity dynamics analysis (FCD; Hutchison et al., 2013; Hansen et al., 2015). This metric has been recently developed to track variability in BOLD connectivity amongst brain areas. Here we will apply this method to EMG signals to track the variability in connectivity amongst muscles. The variability amongst muscles derived from the FCD analysis can be interpreted as the inversed strength of the temporary structural organization between individual muscles, and this variability increases when the muscular organization undergoes a modification. The dynamical systems theory prescribes a peak in the variability of the connectivity of its functional units, one expects to observe a peak in the muscular coordination variability around the transition between behavioral dynamical regimes, in both young and older participants. Concerning the difference of muscular coordination variability between young and older adults, one could predict that older adults will be more variable than young participants. Indeed, classically elderly adults show increased levels of performance variability in both motor and cognitive tasks (Hultsch et al., 2008; MacDonald et al., 2009). This is not to say however that coordination variability observed at muscular level in older adults automatically is expected to be higher than those observed in young adults. For instance, McIntosh and colleagues (McIntosh et al., 2014) showed for their task that while behavioral variability increased with aging, variability observed in brain connectivity decreased. Thus, it might be that variability of coordination patterns measured by FCD reveals similar results.

## Materials and methods

### Participants

Fourteen right-handed older adults [age: 74.4 ± 3.7 years (mean ± *std*), 6 males] volunteered to take part in the experiment. All declared to be in good health and to have a full range of motion with their right upper limb. Throughout this paper, their results will be compared to previously published results of 14 right-handed younger adults [Vernooij et al., 2016; age: 23.2 ± 2.0 years (mean ± *std*), 8 males]. Volunteers did not exercise or consume more than one glass of alcohol within the 12 h preceding participation (confirmed by questionnaire). An informed consent form was obtained from all participants. A local ethics committee approved the study, which was carried out in accordance with the Declaration of Helsinki.

### Experimental details

Experimental design, setup and analysis methods were identical to those reported in Vernooij et al. (2016) to enable direct comparison with young subjects. In short, participants slid a stylus rhythmically over a graphical tablet placed in front and on the right side of the subject between two horizontal bar-targets. The resulting movement was in the sagittal plane to enforce a multi-joint muscular activation. The goal of the task was to move as quickly and accurately as possible between the two bar-targets (i.e., managing the speed-accuracy trade-off). The bar targets were 20 cm apart (A) and could have five different widths (W; 0.33, 0.63, 1.17, 2.18, and 4.06 cm). The index of difficulty (ID) for the five conditions can be calculated as $ID=log2\left(\frac{2A}{W}\right)$. Each of these five IDs were repeated four times, which made 20 trials per subject in total, and each of these trials consisted out of 40 back and forth movement cycles. As the task of the subjects was to move as quickly as possible while staying precise, any trial where subjects made errors in more than 10% of the aiming movements (>8 out of 80 aiming movements per trial) had to be repeated. This forced the subjects to stay focused. After a successful completion of four trials of a certain ID, the width of the bar was altered to change ID. The order of presentation of IDs to the subjects was randomized per block of four trials.

Surface electromyography (EMG) was registered at 1925 Hz (Trigno wireless EMG, Delsys Inc, USA) from the muscle belly of brachioradialis (BR), pronator teres (Pron), biceps brachii short head (BicSho), biceps brachii long head (BicLo), brachialis (Brach), triceps lateral head (TriLat), triceps long head (TriLo), pectoralis (Pect), deltoid anterior (DeltA), deltoid medialis (DeltM), deltoid posterior (DeltP), and teres major (TerM) muscles. To support the electrodes, cohesive bandages (Lastopress 7 cm × 1.5 m, Hartmann Group, Germany) were wrapped around the upper limb. As is classically done when experiments involve EMG data, three maximum voluntary contractions lasting ~5 s each were measured for a pushing and a pulling movement to normalize the EMG signals per subject and to control for muscular fatigue.

### Kinematic data analysis

Further analysis was carried out offline using custom written MATLAB scripts (Mathworks MATLAB 2012b, USA). Positional data, acquired at 250 Hz, were resampled to 100 Hz, cut in cycles based on reversal points of the position and the data of each trial's first two and last three cycles were removed. The movement time (*MT*), acceleration time (*AT*) and deceleration time (*DT*) were calculated per half-cycle and pooled over subjects. The effect of ID on *MT*, *AT*, and *DT* were analyzed using a repeated measures ANOVA with ID as within-subject factor, group (old vs. young) as between-subjects factor and α = 0.05. Where sphericity was violated, the Greenhouse-Geisser adjustment was applied. A Student *t*-test with Bonferroni corrections tested differences between IDs where the main effect of ID was significant.

We studied the *AT*/*DT* ratio for breakpoints to uncover any discontinuity in the kinematics.

A vector field reconstruction for position and velocity was calculated for each trial in order to examine the deterministic components of the system dynamics in 2 dimensions. Concisely, a probability matrix *P*(*x, y, t* | *x*_*o*_, *y*_*o*_, *t*_*o*_) is calculated, which represents the probability of the system to be at state (*x, y*) at time *t* when knowing the current state (*x*_*o*_, *y*_*o*_) at time step *t*_*o*_ (for details, see (van Mourik et al., 2006)). We calculated this probability matrix of the time-series of our position *x*(*t*) and velocity *y*(*t*) over a 99 × 99 equally bin-sized grid for each trial. The deterministic components per bin were calculated as:

$$D_{y}\left(x,\;y\right)=\;\underset{\tau \;\to \;0}{\lim}\;\frac{1}{\tau }\iint \left(x^{\prime }-x\right)\;\;P\;\text{ (}x^{\prime },\;\;y^{\prime },\;\;t\;+\;\tau \;|\;\;x,\;\;y,\;\;t)\;dx^{\prime }\;dy^{\prime }$$

and

$$D_{y}\left(x,\;y\right)=\;\underset{\tau \;\to \;0}{\lim}\;\frac{1}{\tau }\iint \left(y^{\prime }-y\right)\;P\;\;\left(x^{\prime },\;y^{\prime },\;\;t\;+\;\tau \;|\;\;x,\;\;y,\;\;t\right)\;dx^{\prime }\;dy^{\prime }$$

These components identify the system's vector field in the phase space per bin in mathematical terms. Thus, they reconstruct the phase flow, thereby unequivocally determining the dynamical regime of the system. For each bin, we calculated the angle θ between neighboring velocity vectors for the first two deterministic components (i.e., position and velocity). Per reconstruction, the maximum angle (θ_max_) was computed at reversal points of the movement, indicating whether a fixed point existed (θ_max_ ~180°) or not (θ_max_ << 180°) in which case the dynamical regime is described as a limit cycle. We fitted a sigmoid curve through the θ_max_ as a function of ID as $\theta_{\text{max}}=\;\frac{1}{1\;+\;e^{a\left(b+ID\right)}}$. The transition between dynamical regimes was identified as the point of inflection of the sigmoid.

### Muscular variability calculation

We employed functional connectivity dynamics (FCD; Hansen et al., 2015) to study the within-trial variability in muscular control. For each trial, we correlated each of the 12 EMG signals over a two cycle sliding window (N_p_ = 200 time points; MATLAB function corr with option “coef,” full overlap between windows) and subsequently correlated these N_m_ × N_m_ (muscle-by-muscle) matrices to obtain an N_t_ × N_t_ (time-by-time, N_t_ = 3300) FCD matrix showing correlational variability over the course of the trial (see **Figure 2A** for an example of an FCD matrix). We then converted the FCD to a “jump length” matrix as *JL* = 1− *FCD*. This JL signified correlation distance between consecutive time windows. JLs were concatenated over the four repetitions. We estimated the jump length distribution using the 204th diagonal of the FCD matrices (first diagonal which did not include overlap between windows) by calculating the median jump length on this diagonal. This jump length distribution (JLD) captures the statistics of the fluctuations in muscular coordination, therefore representing a measure of variability. An extended explanation of the procedure can be found in Vernooij et al. (2016). The effect of ID on the peak of the distribution was tested with a repeated measures ANOVA with ID as within-subject factor, group (young vs. old) as between-subjects factor and α = 0.05. We used the Greenhouse-Geisser adjustment whenever the assumption of sphericity was violated. Significant effects were subjected to a Student *t*-test with Bonferroni corrections to test for differences between IDs.

### Muscular coordination pattern calculation

Each EMG signal was cut per cycle based on positional data, band-pass filtered (Butterworth zero-time lag, 2nd order, cut-off band: 10–450 Hz), full-wave rectified, and low-pass filtered at 5 Hz (Butterworth zero-time lag, 2nd order) to obtain the envelope per cycle. Then, each envelope was normalized in size in reference to the peak MVC (which was processed the same way) and normalized in time to N_p/c_ = 100 time points. To track changes in the repertoire or use of muscular coordination patterns in detail, we extracted temporarily assembled functional units of coordination (comparable to the previously termed “muscle synergies,” see d'Avella et al., 2003) over small time bins. For each participant and ID, muscle synergies were extracted per 2 cycle bin by applying nonnegative matrix factorization algorithm (NNMF; Lee and Seung, 1999; Tresch et al., 2006; De Marchis et al., 2013) to the EMG matrix *M* of size N_m_ × N_p_ where N_m_ = 12 muscles and N_p_ = 200 time points. NNMF is an iterative optimization method that minimizes the normative error-matrix of ||*M*−*WH*||, where *W* is an N_m_ × N_s_ matrix containing the relative activations of each of the muscles with N_s_ being the number of synergies selected for extraction, and H is an N_s_ × N_p.c_ matrix containing the time-varying activity of each synergy. We extracted 1–12 synergies per NNMF and each NNMF was repeated 100 times (total 1200 NNMFs). The number of *W* chosen for further analysis was defined as the minimum that could explain at least 90% of variance accounted for (*VAF*) (comparable to i.e., Ivanenko et al., 2004; Torres-Oviedo et al., 2006; De Marchis et al., 2013), where

$$VAF=100*(1-\frac{norm{\left(M-WH\right)}^{2}}{norm{\left(M-mean\left[M\right]\right)}^{2}})$$

*W* from all bins and participants were normalized in height and pooled. To discover the number and constitution of the synergies that were used for the task, similar synergies were grouped and group-averaged according to the following procedure. *W* from all bins and all participants were pooled per ID and mapped by performing a 2-dimensional Sammon's mapping (Sammon, 1969). In short, Sammon's mapping plots the set of *W* belonging to a *N*_*m*_ -dimensional space to a set of 2-dimensional vectors while keeping the structure of the *W* intact. This is done by an error minimization function which uses the Euclidean distance between the *W*. Then, the resulting Sammon's map values are subjected to a clustering algorithm (MATLAB function clusterdata with ward's minimum variance method), which quantifies the number of clusters (i.e., groups of similar synergies) that underlie the complete set of *W* per ID. Three to fifteen clusters were calculated for each ID. The minimum number of clusters where the correlation coefficient within each cluster had a median value above 0.5 was selected. *W* were then averaged within each cluster to calculate the representative synergies (${W_{cluster}}^{\prime }s$) per ID.

As it turned out that the $W_{cluster}{\left(ID\right)}^{\prime }s$ were relatively similar between IDs, we averaged the ${W_{cluster}}^{\prime }s\;$ over IDs (*W*_*basis*_).

We used two methods to discover a reorganization of muscular coordination patterns, i.e., a change in the use of the *W*_*basis*_: (1) the number of times each *W*_*basis*_ was extracted was identified per ID; (2) a principal component analysis (*PCA*_*syn*_) was applied on the concatenated matrix of *H* per ID. This gave us insight in the use of the synergy-repertoire and the variability in the amplitude of each *W*_*basis*_ over time. Additionally, we calculated Pearson's correlations between *W*_*basis*_ found for young subjects with those found in elderly subjects to determine how any age-related change in coordination is expressed in type of muscular assembly.

## Results

### Qualification of behavioral dynamical regimes and kinematics

The repeated measures ANOVA on the kinematic data showed a significant Age^*^ID interaction [*F*_(2.42, 266.20)_ = 9.61, η^2^ = 0.08, *p* < 0.000; *F*_(2.76, 303.65)_ = 3.24, η^2^ = 0.029, *p* = 0.026; *F*_(2.21, 242.90)_ = 12.96, η^2^ = 0.11, *p* < 0.000 for *MT*, *AT*, and *DT* respectively]. We thus looked at the effect of ID separately for the elderly adults. For elderly adults, as was the case for young adults, *MT*, *AT*, and *DT* increased monotonically with ID [*F*_(2.24, 123.19)_ = 435.56, η^2^ = 0.89; *F*_(2.84, 156.03)_ = 285.70, η^2^ = 0.84; *F*_(2.10, 115.70)_ = 351.09, η^2^ = 0.87; respectively, All *p* < 0.001]. *Post-hoc* analyses showed that all IDs were significantly different from each other for all three variables (all *p* < 0.001). All movement times were significantly higher for elderly adults compared to young adults; the repeated measures ANOVA showed a main effect of age [*F*_(1, 110)_ = 103.66, η^2^ = 0.49; *F*_(1, 110)_ = 55.76, η^2^ = 0.34; *F*_(1, 110)_ = 102.41, η^2^ = 0.48 for *MT*, *AT*, and *DT* respectively, All *p* < 0.001]. *Post-hoc* analyses showed that elderly subjects were significantly slower for all IDs (all *p* < 0.001). In contrast to what was found in young adults, for elderly adults we did not find a breakpoint in the *AT*/*DT* ratio.

**Figure 1:**
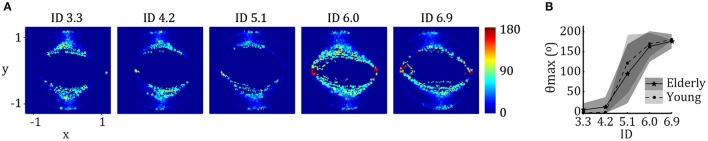
**(A)** Reconstructed angle diagrams as a function of ID averaged across participants. The horizontal axes represent normalized position (*x*); the vertical axes normalized velocity (*y*). The color coding (right side of the panel) represents the maximum angle in degrees between adjacent vectors. Red areas indicate the existence of locally opposing angles and imply the presence of a fixed point. Its absence implies the existence of a limit cycle. **(B)** The maximum angle in degrees between adjacent vectors as a function of ID averaged across participants (θ_max_) for both young (light gray and dotted line) and older (dark gray and solid line) adults. The horizontal axis represents ID; the vertical θ_max_ (degrees). Error bars denote 1 standard deviation.

Figure 1A depicts the average angles (θ) between flows of the vector field reconstruction for elderly adults, where a fixed point can be assumed wherever θ ~180° and a limit cycle wherever θ << 180°. Figure 1B shows that θ_max_ at reversal points is close to 0°For lower IDs and ~180°For higher IDs. The results for young adults, calculated in a previous study, are superimposed. The repeated measures showed a main effect of Age [*F*_(1, 110)_ = 6.10, η^2^ = 0.053, *p* = 0.015] and an Age^*^ID interaction [*F*_(1.79, 196.72)_ = 3.33, η^2^ = 0.029, *p* = 0.043]. *Post-hoc* analysis revealed that elderly show a significantly higher θ_max_ in general, with a significantly higher θ_max_ for ID 4.2 (*p* < 0.000). The inflection point of the fitted sigmoidal curve for elderly is located on average at 5.11 ± 0.42 bits. The repeated measures ANOVA confirms this finding by separating the IDs in three groups; ID 3.3 & ID 4.2 < ID 5.1 < ID 6.0 & ID 6.9 (all *p* < 0.000). This inflection point was not significantly different between the two age groups.

### Variability of muscular coordination

**Figure 2:**
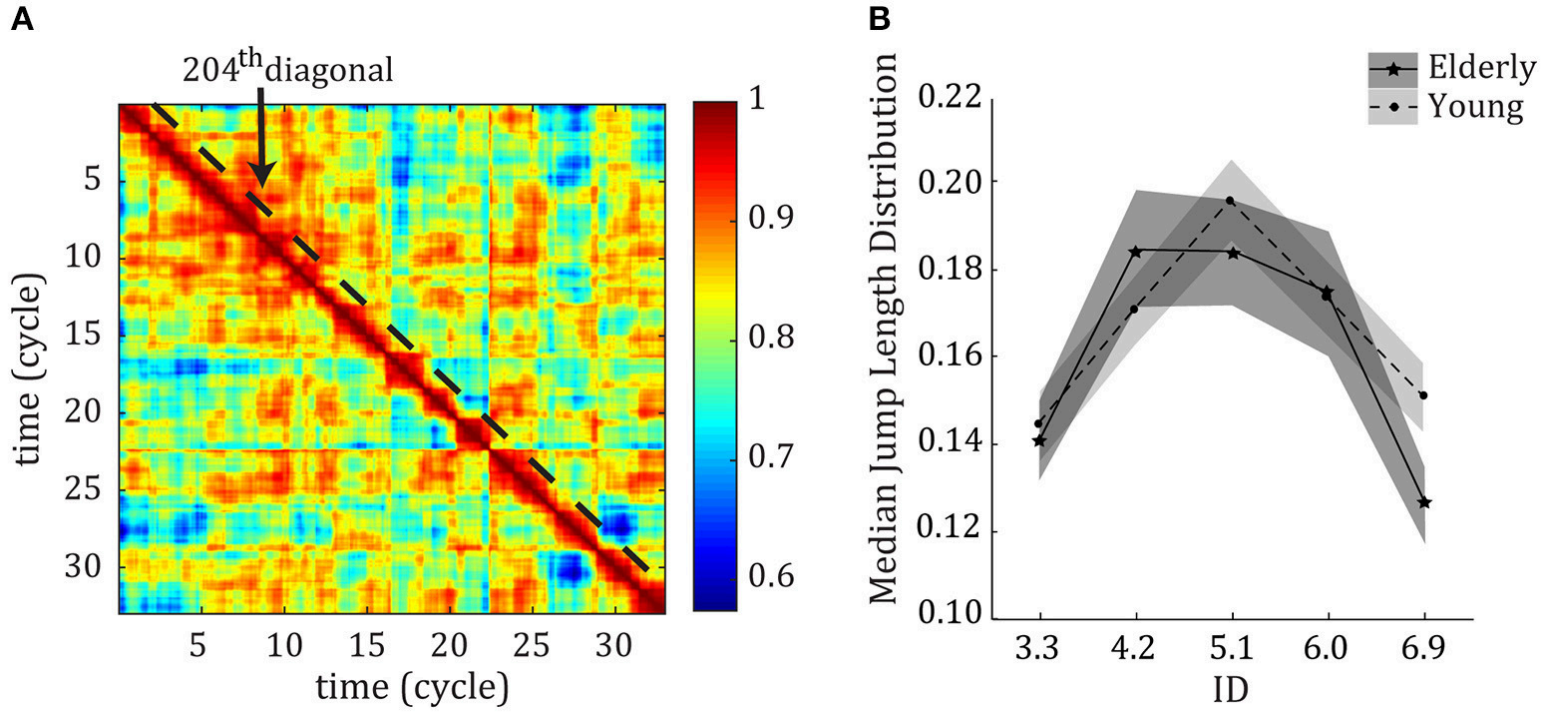
**(A)** Example of an FCD matrix, including the 204th diagonal over which the data was analyzed. **(B)** Median Jump length distribution (*JLD*) curve of the FCD per ID across young (dotted line) and older (solid line) participants *P* < 0.05. Error bars denote 1 standard deviation.

We applied the FCD analysis to the measured EMG signals to study variability in muscular activations (see Figure 2A for an example of an FCD matrix). The median jump length (*JLD*) of the FCD showed that there were larger fluctuations in correlational patterns among muscles at the ID where behavior showed a transition between limit cycle and fixed-point regimes (see Figure 2B). The repeated measures ANOVA showed a main effect of ID on the size of the jumps in muscular coordination [*F*_(3.32, 182.52)_ = 15.18, η^2^ = 0.22, *p* < 0.001]. *Post-hoc* analyses showed that the median *JLD* for IDs 4.2, 5.1, and 6.0 were significantly larger than for IDs 3.3 and 6.9 (all *p* < 0.001).

Although there was no main effect of age (*p* = 0.36), the repeated measures ANOVA showed a trend toward an Age^*^ID interaction [*F*_(3.487, 383.62)_ = 2.38, η^2^ = 0.02, *p* = 0.060]. Interestingly, for older adults the variability was the highest at ID 4.2. For younger subjects, the variability of ID 5.1 was significantly larger than those of all other IDs. Compared to younger adults (tested in an earlier study—results superimposed in Figure 2B) the current results for elderly adults thus show less levels of variability (2 levels vs. 3 levels), and a larger and earlier period of reorganization.

### Muscular coordination patterns

**Figure 3:**
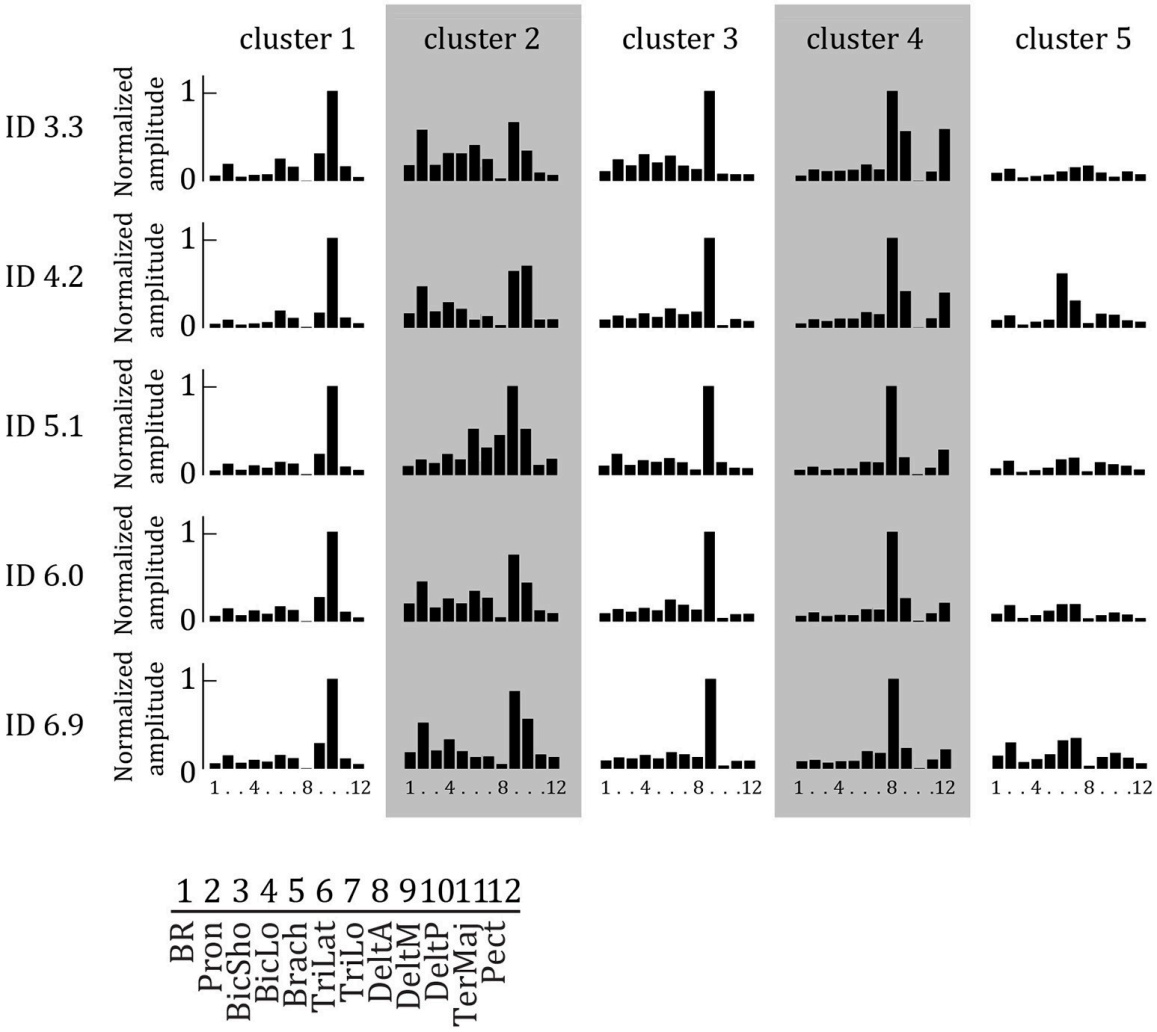
**Synergy weights extracted by NNMF as a function of ID averaged per cluster**. Per synergy, the normalized weight of each of the 12 muscles is represented. Each muscle is activated in multiple synergies. Higher bars indicate increased weight.

We extracted two to seven muscle synergies (*W*) per set of EMG signals recorded during two cyclic behavioral episodes of the elderly subjects to explain at least 90% of the total variance in the EMG data of the 12 muscles. In general, more synergies were extracted for higher IDs (mean ± *std*: 3.49 ± 0.81, 3.68 ± 0.88, 3.53 ± 1.00, 3.71 ± 1.01, 3.77 ± 1.05, respectively). Per ID, Sammon's mapping and the cluster procedure identified five main synergies. These were relatively similar between IDs, and arranged accordingly in Figure 3 to form five *W*_*cluster*_ 's.

**Figure 4:**
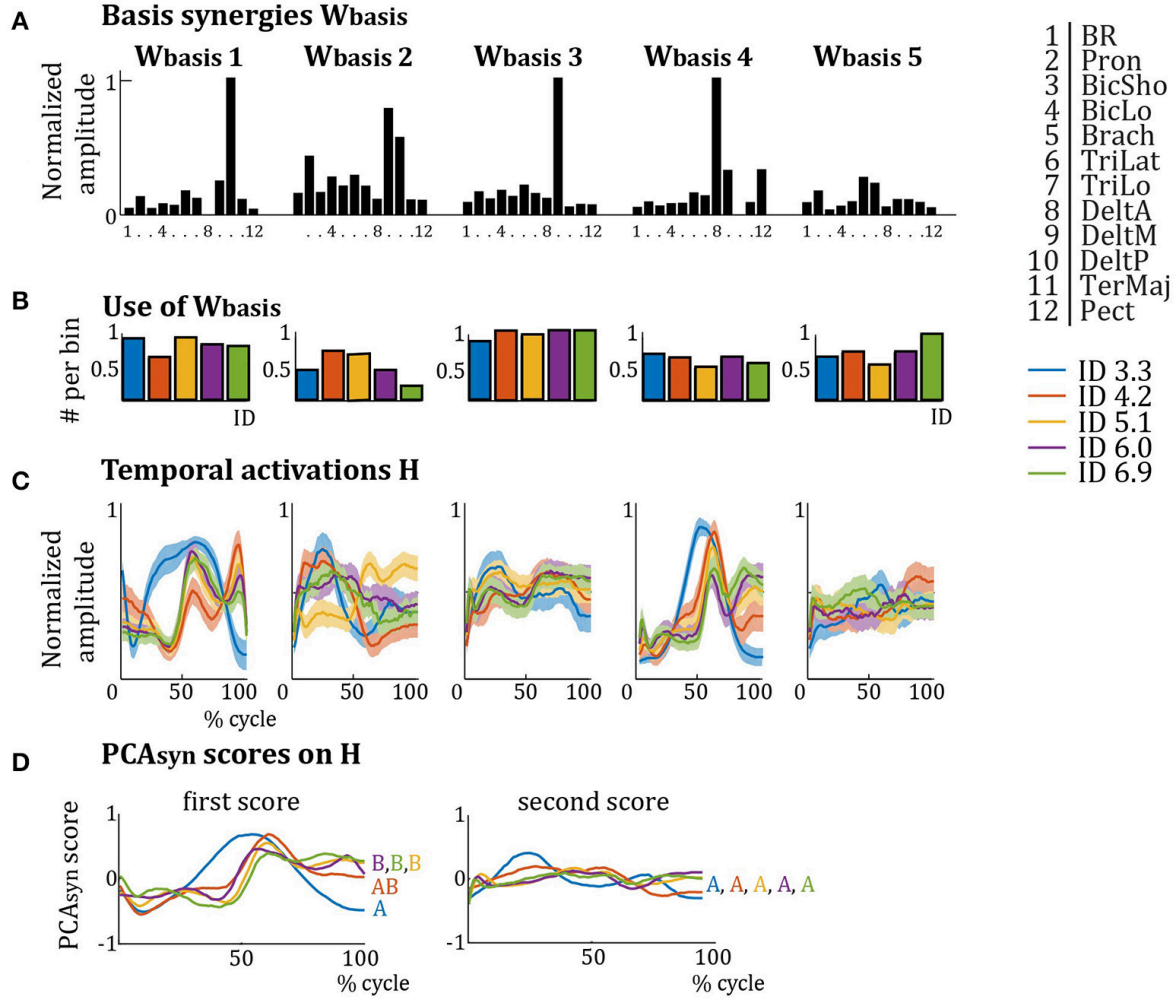
**(A)** Synergy weights from Figure 3 averaged per cluster (*W*_*basis*_). Per basis synergy, the normalized weight of each of the 12 muscles is represented. Higher bars indicate increased weight. **(B)** Normalized frequency of use of the basis synergies per ID. **(C)** Average temporal activation components over a movement cycle per basis synergy per ID. Darker colors represent lower IDs. **(D)** First and second *PCA*_*syn*_ scores on the temporal activations *H* of **(B)**. The first *PCA*_*syn*_ score shows a clear difference between the activations of the IDs, activations with significant correlations (*p* << 0.001) are grouped by letters **(A,B)**, whereas the second *PCA*_*syn*_ score does not (all activations are correlated with all others).

As these *W*_*cluster*_ were similar over IDs, they were averaged per cluster, giving *W*_*basis*_ (see Figure 4). Most muscles are activated in multiple *W*_*basis*_ and most *W*_*basis*_ activate multiple muscles (see Figure 4A). The relative activation of each muscle is different in each *W*_*basis*_. *W*_*basis*_1 mainly activates Pron, TriLat, DeltM, and DeltP; *W*_*basis*_2 mainly activates Pron, biceps and triceps, DeltM, and DeltP; *W*_*basis*_3 mainly activates DeltM; *W*_*basis*_4 mainly activates DeltA, DeltM, and Pect; and *W*_*basis*_5 mainly activates TriLat and TriLo. The results for the use of the *W*_*basis*_ repertoire (Figure 4B) show that elderly subjects recruit *W*_*basis*_2 most around ID 4.2 and ID 5.1, whereas *W*_*basis*_4 and *W*_*basis*_5 are least recruited around ID 4.2 and ID 5.1. Interestingly, the differences in recruitment are much less pronounced for elderly subjects compared to young subjects (Vernooij et al., 2016). *PCA*_*syn*_ analyses on the temporal activation profiles *H* of the elderly subjects (Figure 4C) showed that the score of the first component explained most of the variance between IDs: 74.3, 71.4, 84.7, 84.9, and 90.5%, for increasing IDs respectively. Scores of *PCA*_*syn*_ components two to seven explained less than 20% of variance and were similar per ID (see Figure 4D for *PCA*_*syn*_ scores of the first and second components). This first component differed for ID 3.3, especially around the target approach (40–50 and 90–100% of cycle). Pearson's correlation of scores of the first *PCA*_*syn*_ components showed that the scores for ID 3.3 and ID 4.2 were significantly correlated (*r* > 0.60, *p* << 0.001) and the scores for ID 4.2, ID 5.1, ID 6.0, and ID 6.9 were significantly correlated (all *r* > 0.60, all *p* << 0.001). The score of ID 3.3 was not significantly correlated to those of ID 5.1, ID 6.0, and ID 6.9 (average *r* = 0.17, average *p* = 0.17). Accordingly, the first *PCA*_*syn*_ scores differentiated two types of temporal activation (indicated by letters A and B in Figure 4D), where the switch between the types is at ID 4.2. Pearson's correlations of scores of the second *PCA*_*syn*_ components were all significant (*p* << 0.001), except for ID 3.3 and ID 6.9 which were not significantly correlated (*r* = 0.51, *p* = 0.13). This indicates its lack in explaining much difference between *H* over IDs.

**Figure 5:**
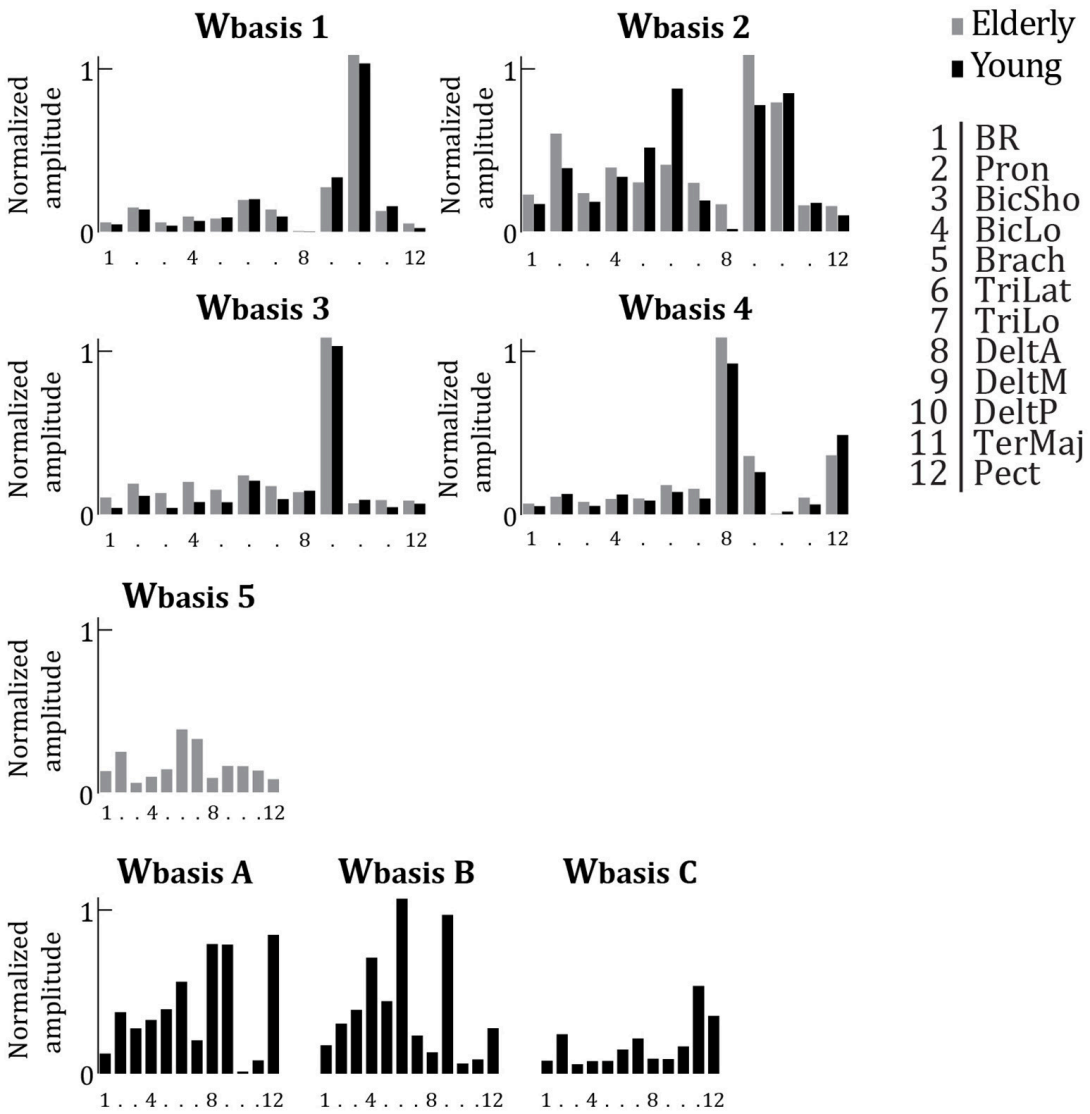
**Averaged synergy activation coefficients (***W***_***basis***_), replotted from Figure 4 (older adults, gray bars) and replotted from Vernooij et al. (2016) (young adults, black bars)**. Per basis synergy, the normalized activity of each of the 12 muscles is represented. Higher bars indicate increased strength of activity. Synergies are grouped based on correlation. Those synergies which were not significantly correlated to a synergy from the other age group are plotted separately (*W*_*basis*_ 5 and *W*_*basis*_ *A, B*, &* C*).

Compared to young adults, elderly have a reduced and slightly modified repertoire of muscular activation patterns. Figure 5 depicts the *W*_*basis*_ of elderly adults (*W*_*basis*_1−5 copied from Figure 4) partly matched with the *W*_*basis*_ of young adults. Additionally, the additional *W*_*basis*_ calculated for young adults which were not correlated to a *W*_*basis*_ calculated for elderly adults are shown in Figure 5 (*W*_*basis*_*A*−*C*). The seven *W*_*basis*_ calculated for young adults seem to comprise four of the *W*_*basis*_ of the elderly adults (all *r* > 0.77, all *p* < 0.003). In other words, four out of seven *W*_*basis*_ identified from the data of young adults match to four out of five *W*_*basis*_ for the elderly. There are however some modifications visible. Where in *W*_*basis*_2 elderly seem to show more activation for DeltM, they appear to activate TriLat much less. Moreover, *W*_*basis*_5 was not significantly correlated to any of the *W*_*basis*_ of the young subjects (all *r* < 0.48, all *p* > 0.12).

In terms of temporal activation of the *W*_*basis*_, the first component of *PCA*_*syn*_ seems to be able to capture the main patterns per ID in both age groups. For both young and older adults, there are two types of main temporal activation patterns distinguishable. These types of temporal activation are similar between the two age groups, but with aging, the switch between the two types occurs at a lower ID (ID 4.2 vs. ID 5.1).

## Discussion

This study aimed at identifying the effect of aging on the muscular dynamics and its relationship to behavioral dynamics. The main issues addressed in this respect were: (1) Are behavioral dynamics observed in Fitts' task in young and older adults comparable? (2) Does aging affect the muscular coordination dynamics associated with behavioral dynamics? To answer these questions, we manipulated task difficulty via target width in a unimanual, cyclical Fitts' task performed by older adults in order to provoke a change in their behavioral regime. In addition to behavioral data, we measured the surface EMG of 12 arm- and shoulder muscles to identify muscular coordination patterns (synergies) and variability in muscular coordination. Results were compared with those previously observed in young adults (Vernooij et al., 2016).

### Qualification of behavioral dynamical regimes and endpoint kinematics

At kinematic level, *MT*, *AT*, and *DT* significantly increase with higher ID and older adults in general had larger movement times than younger adults. These results are consistent with those reported in previous studies using discrete Fitts' tasks (e.g., Temprado et al., 2013; Sleimen-Malkoun et al., 2013). In contrast to results observed for young adults (Huys et al., 2010, 2015; Vernooij et al., 2016), we did not find a breakpoint in the *AT*/*DT* ratio for elderly adults. A similar result was previously observed in discrete Fitts' task (Sleimen-Malkoun et al., 2013). The absence of a breakpoint suggests that older adults used a similar kinematic organization whatever the accuracy constraints. This is consistent with the dedifferentiation hypothesis (Baltes et al., 1980; Baltes and Lindenberger, 1997) at kinematic level, which conjectures that with aging there is a loss of the specialization, and systems become simplified, less distinct or common to different functions.

Our results also showed that older adults presented a transition in behavioral dynamics with increasing ID. Indeed, phase flow analyses on the reversal points of the movement displayed a switch in difference in flow angle from ~0° to ~180° with higher IDs, which is associated with a transition from limit-cycle to fixed-point. As for younger adults, this transition occurred on average at an ID ~5.1. However, as supported by the significant Age^*^ID interaction, older adults seemingly “started” the reorganization at a lower ID. That is, in older adults a percentage of movements performed at ID 4.2 might already show a fixed-point regime, whereas this was not the case for younger adults. To verify this hypothesis, one should calculate the distribution of movements performed as limit-cycle and fixed-point for each ID. Unfortunately, there is no technique currently available to scientifically test changes in the distribution of dynamical regimes per trial in Fitts' task. Phase flow analyses require many repeats of the same movement to reliably determine the flow of the movement, and thus do not allow identification of dynamical regime per cycle. Overall, the present results observed in Fitts' task are comparable to those observed by Temprado et al. (2010) in a bimanual coordination task. They have shown comparable behavioral dynamics between young and older adults with a phase transition occurring at a slightly lower ID in older adults.

In the present context, the absence of a breakpoint in *AT*/*DT* ratio in combination with the transition in behavioral dynamics (as indicated by phase flow analyses) is surprising since in previous studies changes in kinematic patterns and in dynamical regimes were considered to be closely related (Huys et al., 2010, 2015; Sleimen-Malkoun et al., 2013; Vernooij et al., 2016). The comparison between kinematic patterns and dynamical regimes in young and older adults suggest that these two levels of description of behavior are not systematically related, at least in older adults. Consequently, one should be careful with the interpretation of kinematic data in the context of coordination dynamics in the absence of knowledge about behavioral dynamics by means of the use of specific tools (e.g., phase flow analyses).

### Change in variability of muscular coordination

Our main interest in this study was to track how aging affects muscular dynamics in relationship to a transition in behavioral dynamics. Accordingly, we calculated a measure of muscular coordination variability, obtained from Functional Connectivity Dynamics analyses (FCD; Hansen et al., 2015), to examine the presence of increased variability around the behavioral transition and we compared this profile to variability found in young adults.

Consistent with results obtained for younger adults for Fitts' task (Vernooij et al., 2016), muscular coordination of older adults show increased levels of variability around the behavioral transition in dynamical regime. Specifically, the FCD carried out on the EMG data showed increased muscular variability for ID 4.2, ID 5.1, and ID 6.0 compared to ID 3.3 and ID 6.9. This suggests that phase transition in behavioral dynamics is accompanied by larger switches between assemblies of the functional components of the muscular system (i.e. the muscles). Interestingly, for older adults muscular variability is highest at ID 4.2. This is in line with the behavioral results, which suggest that the change in dynamical regime starts at a lower ID than in young adults. Taken together with the similar level of variability for ID 4.2, ID 5.1, and ID 6.0, this also suggests that the reorganization of the muscular system is plateaued in older adults compared to young participants.

Compared to younger adults, variability in muscular coordination does not change as much for elderly. For younger adults, muscular coordination showed a significantly increased variability between ID 3.3 and ID 4.2 and between ID 4.2 and ID 5.2, after which the variability decreased again significantly with each higher ID. In contrast, variability of older adults in the intermediate three IDs was similar, and they were together significantly more variable than the extreme high and low IDs. The effect of age on variability over IDs was nearly significant (Age^*^ID interaction *p* = 0.06). This could suggest that older adults tend to less flexibly coordinate their muscular system to accommodate the transition in behavioral dynamics.

Here the question is whether the FCD results for older adults reflect less switching across a comparable number of states in the repertoire or whether it reflects less switching due to a decrease in the number of available states in the repertoire of muscular coordination patterns. In other words, the results might suggest that the set of dynamical repertoires used is the same between age groups but elderly do not switch between them as often or alternatively that a smaller set of dynamical repertoires is available. Only the latter case would be consistent with the dedifferentiation hypotheses (Sleimen-Malkoun et al., 2014) which was also found for the kinematic data presented here. The analyses of muscular synergies allowed answering this question.

### Reorganization of muscular coordination patterns

In line with results observed for young participants, the NNMF analysis (Lee and Seung, 1999; Tresch et al., 2006; De Marchis et al., 2013) showed that the 12 recorded muscles were not activated randomly across IDs. Instead, they were assembled based on a limited repertoire of synergies which captured muscular coordination used to perform the task. Specifically, the NNMF analysis indicated (i) that the muscular coordination of elderly subjects could be explained by only five synergy patterns, and (ii) that these synergy patterns were similar between IDs. In other words, five muscular synergies are sufficient to describe muscular coordination in Fitts' task, thereby suggesting a significant dimensional reduction of the muscular system independent of the ID. This result is consistent with those observed in previous studies examining different tasks (e.g., walking: Cappellini et al., 2006; reaching: d'Avella et al., 2008; gait transitions: Hagio et al., 2015; cycling: De Marchis et al., 2013). However, here we showed that (i) a change in the behavioral dynamics resulting from increasing accuracy constraints is associated with muscular reorganization, based on PCA analyses of the temporal activation patterns, and (ii) the repertoire of muscular activation patterns is modified and reduced over aging.

Whatever the repertoire of muscular patterns used to perform Fitts' task across ID, we predicted to observe a different assembly of (the same) muscular patterns around any behavioral transition. The results confirmed this prediction. We observed a reorganization of the temporal activations *H* for elderly with higher ID. PCA analyses on *H* showed that the first component of *PCA*_*syn*_, explaining 70–90% of the temporal activation profiles, is significantly altered at ID 4.2. As *H* for high IDs were not similar to the profiles for low IDs, it strongly suggests there is a distinction in temporal activation of the synergies between high and low IDs. The switch between the *PCA*_*syn*_ at ID 4.2 can be interpreted as a reorganization of the muscular system. This reorganization coincides with the peak variability calculated by the FCD analyses. The reorganization, again, indicates that older adults start their reorganization at a lower ID than young adults do (old adults at ID 4.2, and young adults at ID 5.1). The appearance of alterations in temporal activation profiles only has been shown during phase transitions in gait (Ivanenko et al., 2004; Cappellini et al., 2006; Hagio et al., 2015). It can therefore be suggested that a phase transition in behavioral dynamics is initiated by a reorganization of the muscular system by means of altered phasing of muscular coordination patterns. Note that a mere parametrization of the synergies or its temporal activation does not constitute a true reorganization; only a change in the structure or number of synergies or their activation profiles would signify a reorganized muscular coordination. As we normalized our data in time and amplitude, we abolished any parametrization effect and any change presented thus represent some degree of reorganization.

The subsequent comparison of the repertoire of muscular patterns observed in young and older adults showed a decrease in the number of synergy required to capture the organization of the muscular system in older adults (from 7 in young to 5 in older participants). This reduction of the repertoire of patterns seems to be a general principle of aging. Indeed, it has already been observed in previous studies for kinematic patterns (Sleimen-Malkoun et al., 2013). Our findings suggest that this principle also applies at muscular level, although they are not consistent with those reported by Monaco et al. (2010). These authors presented no reduction in the number of synergies used by older adults during locomotion. However, since they preset the number of synergies to extract, any conclusion on reduction of repertoire based on their study is misleading. It must also be noticed that the reduction of the synergy repertoire we reported for elderly adults is unlikely to be due to the lower movement velocity recorded in elderly adults seeing that there is a large overlap in movement velocities between young and older adults. Additionally, previous studies on different aiming velocities have not found a reduction in synergy repertoire (d'Avella et al., 2008).

Our analyses also indicated that the muscular synergies used by young and older adults were only partly similar. Among the seven synergies used by young participants, four of them were common to the ones used by older adults, but the three others were specific to young subjects (see Figure 5). Interestingly, two *W*_*basis*_ (main synergy weight structures) calculated for young adults, but not for elderly adults, were those that showed a clear peak or a clear valley in the recruitment around the behavioral phase transition of young adults. Together, this may indicate that older adults lose those coordination patterns that are associated with a change in organization, which would be related to a decreased flexibility in coordination of the muscular system. Additionally, the variation in recruitment of synergies over IDs seems to be much less pronounced for older adults compared to younger adults. These results suggest that the dimensional reduction in muscular coordination is constrained by the task (external constraint) and by changes in the neuro-musculo-skeletal system (internal constraint).

## Author contributions

CV, GR, FR, and JT. contributed to the conception of the study, FR helped with recruitment and screening of subjects, CV carried out the experiment and performed the data analysis, CV, GR, and JT. wrote and/or reviewed the manuscript. FR and EB approved submission. All persons designated as authors qualify for authorship, and all those who qualify for authorship are listed.

### Conflict of interest statement

The authors declare that the research was conducted in the absence of any commercial or financial relationships that could be construed as a potential conflict of interest.
